# Persistent falcine sinus in the newborn: 3 case reports of associated anomalies

**DOI:** 10.1016/j.radcr.2022.11.019

**Published:** 2022-12-22

**Authors:** Lee K. Rousslang, Tanner J. Coleman, Jaren T. Meldrum, Dustin Roberie, Veronica J. Rooks

**Affiliations:** a Department of Radiology, Tripler Army Medical Center, Medical Center, Honolulu, 1 Jarrett White Rd Medical Center, HI 96859, USA; b Department of Radiology, Alaska Native Medical Center, Anchorage, AK, USA

**Keywords:** Falcine sinus, Pediatric neuroradiology, Vein of Galen, Parietal cephalocele

## Abstract

The falcine sinus is a normal embryonic structure that is situated between the 2 layers of the falx cerebri and drains the deep cerebral venous system into the superior sagittal sinus. It normally involutes after birth and is uncommon in adults. Although it is often an isolated and incidental finding, it can also be associated with a number of other conditions including but not limited to vein of Galen arterial malformations (VGAM), atretic parietal cephaloceles, acrocephalosyndactyly (Apert syndrome), absence of the corpus callosum, absence of the tentorium, osteogenesis imperfecta, or Chiari II malformations. We present a case series of 3 pediatric patients born with a persistent falcine sinus and an associated condition, including a VGAM, an APC, and a sinus thrombosis. The purpose of this article is to highlight the importance of understanding anatomic variations in the cerebral venous system to help aid in the proper diagnosis and treatment of associated pathologies.

## Introduction

**Fig. 1:**
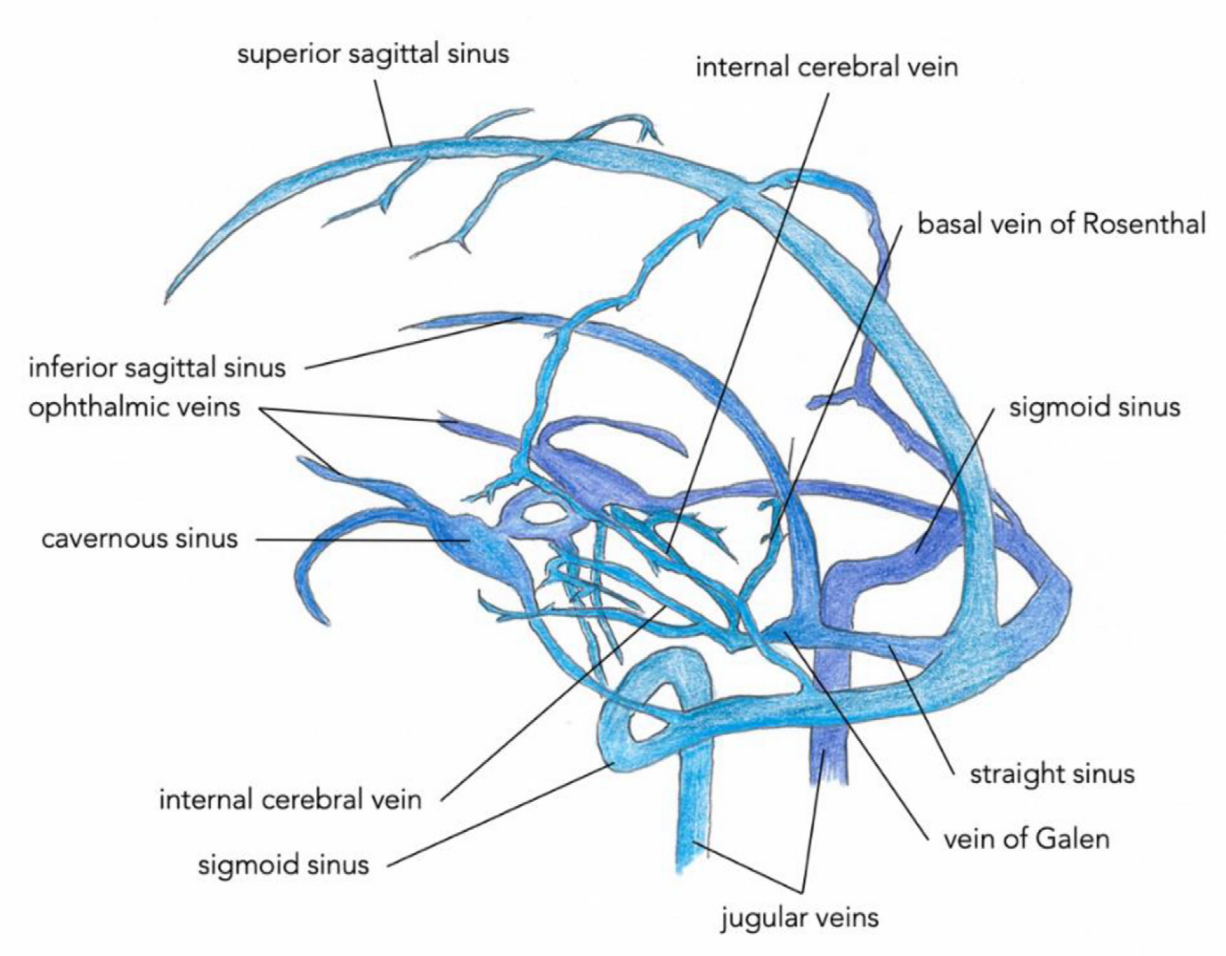
Normal cerebral venous anatomy.

The falcine sinus is located in the falx cerebri and drains the posterior vein of Galen of the deep cerebral venous system to the superior sagittal sinus in the fetus (Fig. 1). It normally closes after birth.

**Fig. 2:**
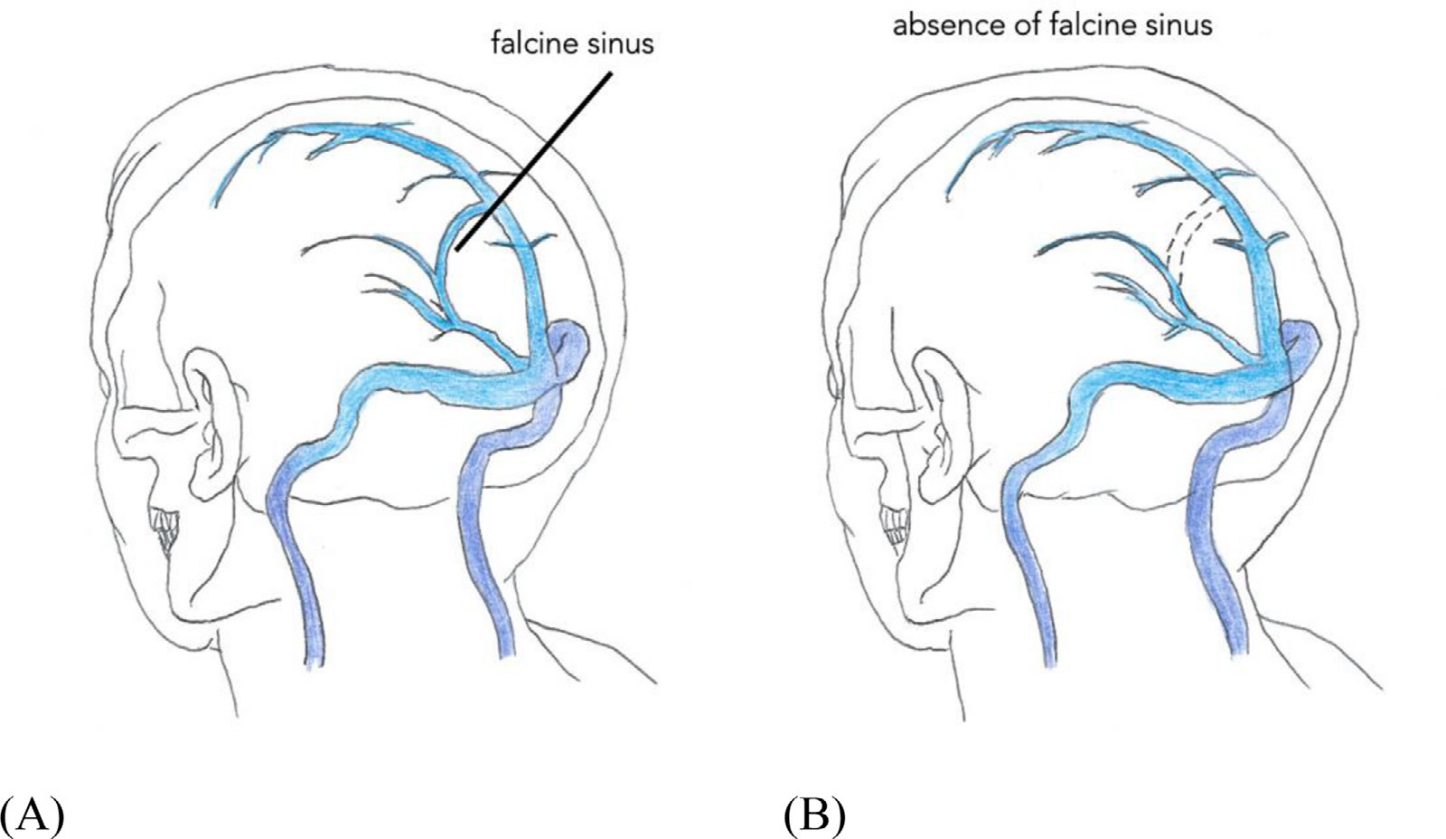
(A) The sagittal plexus joins the tentorial plexus. (B) There are continuous alterations and adjustments of the venous plexiform as the cerebellum grows.

During embryonic development, the primitive falx cerebri contains a mesh of anastomotic venous loops known as the sagittal plexus. One of the caudal anastomotic loops of the sagittal plexus gives rise to the falcine sinus, which connects the posterior vein of Galen with the superior sagittal sinus in the developing fetus. The falcine sinus normally disappears after development of the superior sagittal sinus and straight sinus are complete [1] (Fig. 2). If the straight sinus fails to develop, the falcine sinus is thought to persist to enable venous drainage from the deep cerebral system [2].

Persistent falcine sinus is not an uncommon incidental finding in pediatric patients. In a retrospective review of 3135 pediatric brain MRIs completed at Penn State Hershey Medical Center between January 2008 and December 2009, 1% of patients were found to have a persistent falcine sinus [3]. It can be seen with conditions such as vein of Galen arterial malformation (VGAM), arteriovenous malformations, absent corpus callosum, osteogenesis imperfecta, or Chiari II malformations [2].

## Case series

### Case 1: Vein of Galen arterial malformation (VGAM)

**Fig. 3:**
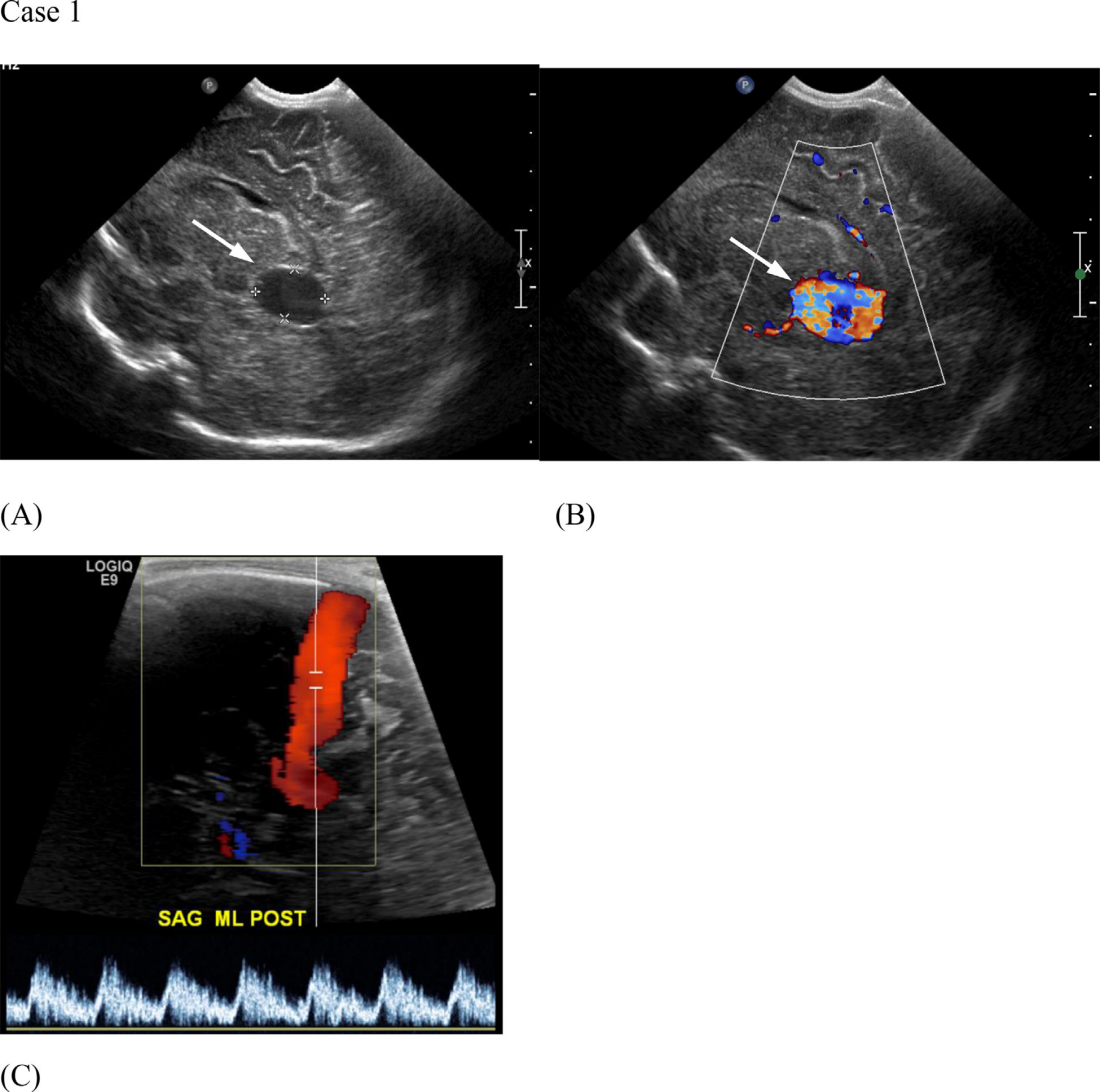
(Case 1) Head US without (A) and with (B) color Doppler demonstrate a vein of Galen malformation (arrows). Spectral Doppler (C) demonstrates a persistent falcine sinus extending superiorly from vein of Galen malformation.

**Fig. 4:**
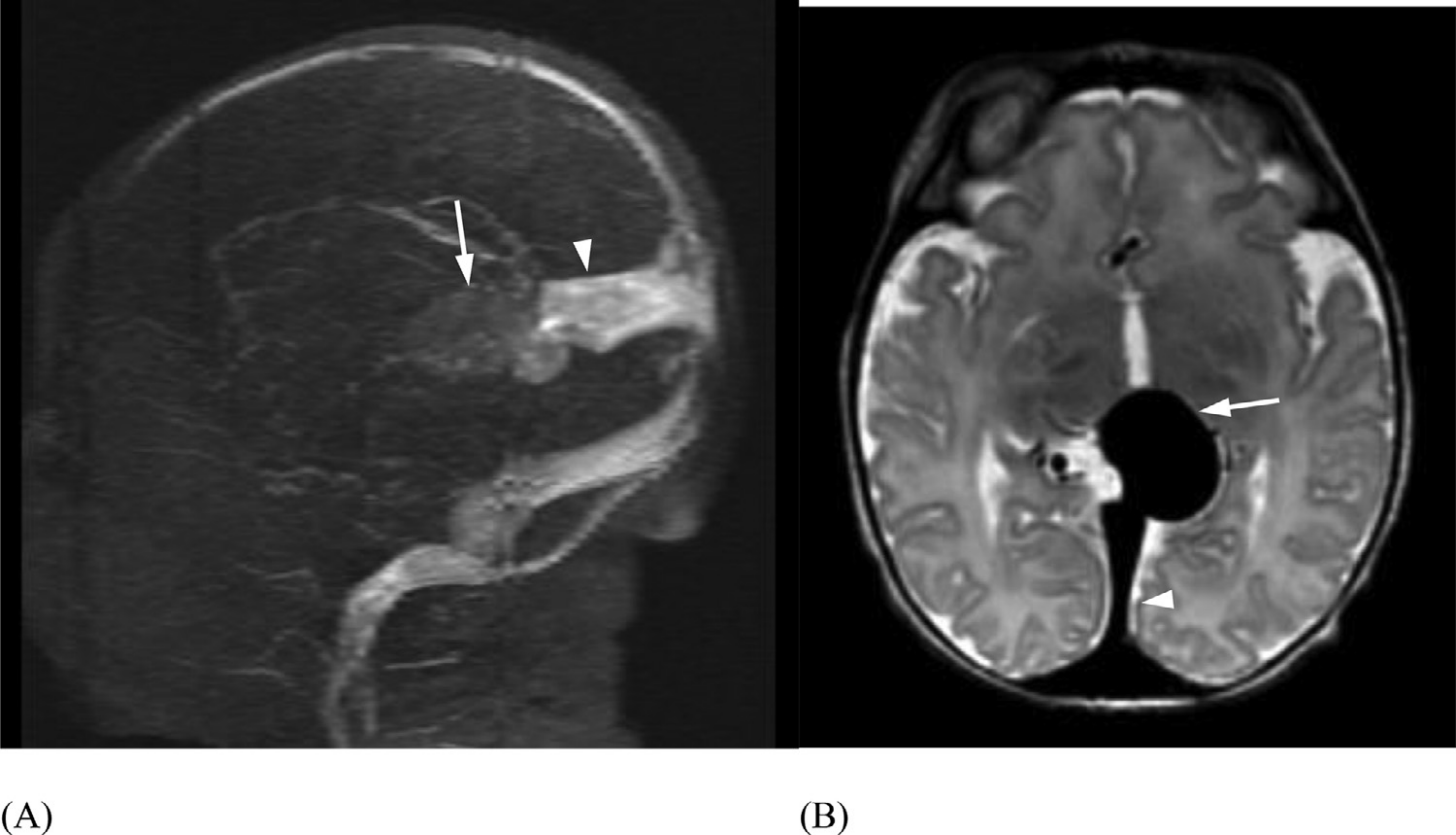
(Case 1) MRV (A) and axial MR (B) demonstrating a persistent falcine sinus (arrowheads) draining the median prosencephalic vein (arrows).

In her first week of life, a baby girl born with a history of VGAM discovered in utero presented for a follow up neonatal head ultrasound. Ultrasound redemonstrated the VGAM, as well as a persistent falcine sinus (Fig. 3). Same day MRI was performed for further evaluation of the VGAM (Fig. 4). By 2 months of age, she developed subclinical high-output heart failure with left ventricular enlargement secondary to her VGAM, which was controlled medically. By 5 months of age, she required escalating doses of furosemide (Lasix) and digoxin (Lanoxin) to control her heart failure. No hydrocephalus was present. Intervention in the form of endovascular embolization was deemed necessary to prevent fulminant heart failure.

Interventional embolizations were performed in stages to reduce the risk of thrombosis or hemorrhage. By 21 months old, the patient underwent 6 arterial embolizations, which appeared to be curative. As of age 5, the patient appears to be doing very well developmentally and intellectually. She has no cognitive or neurological problems and is meeting all developmental milestones.

### Case 2: Atretic parietal cephalocele (APC)

**Fig. 5:**
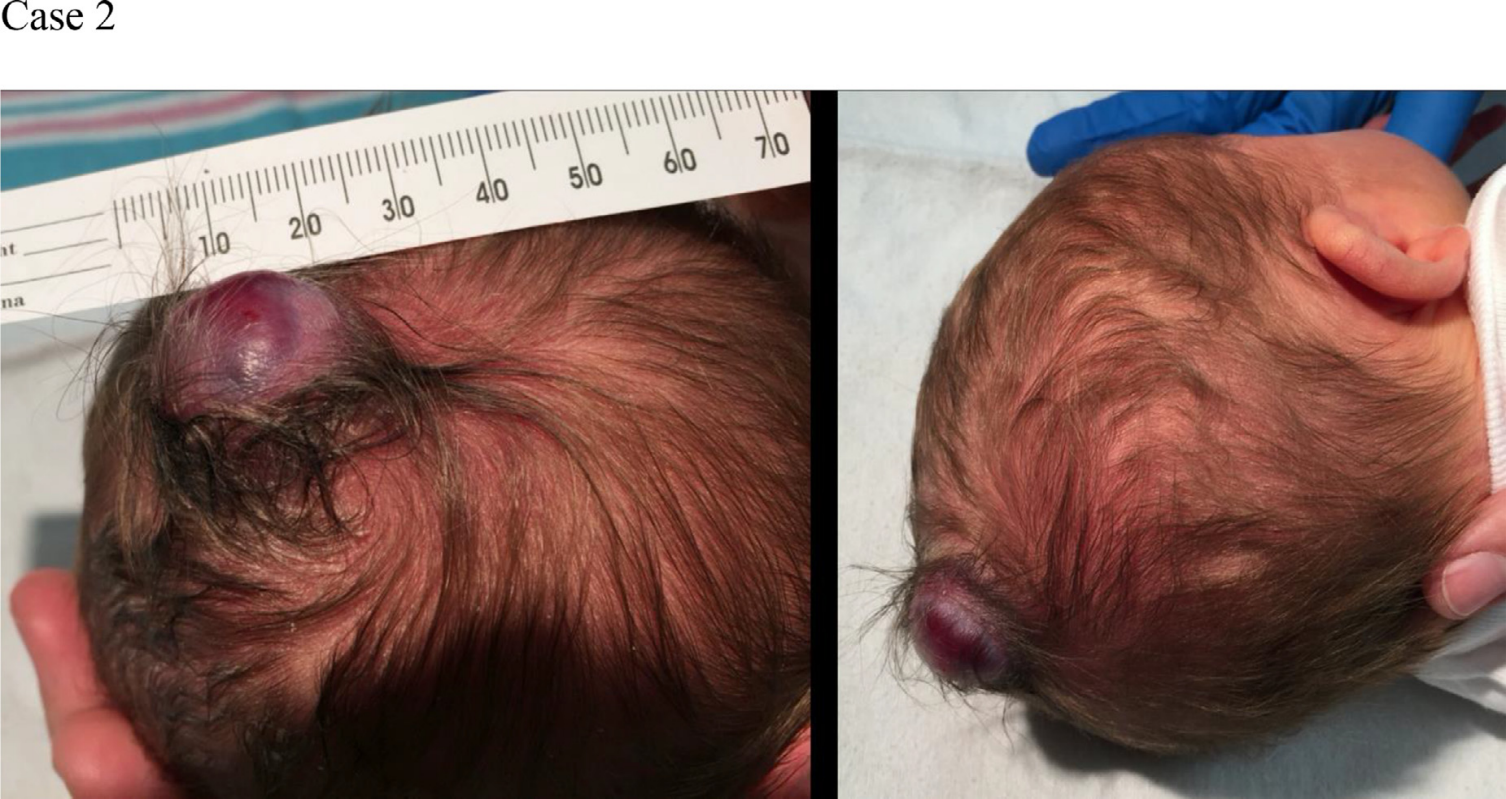
(Case 2) Small subscalp lesion in patient with atretic parietal cephalocele.

**Fig. 6:**
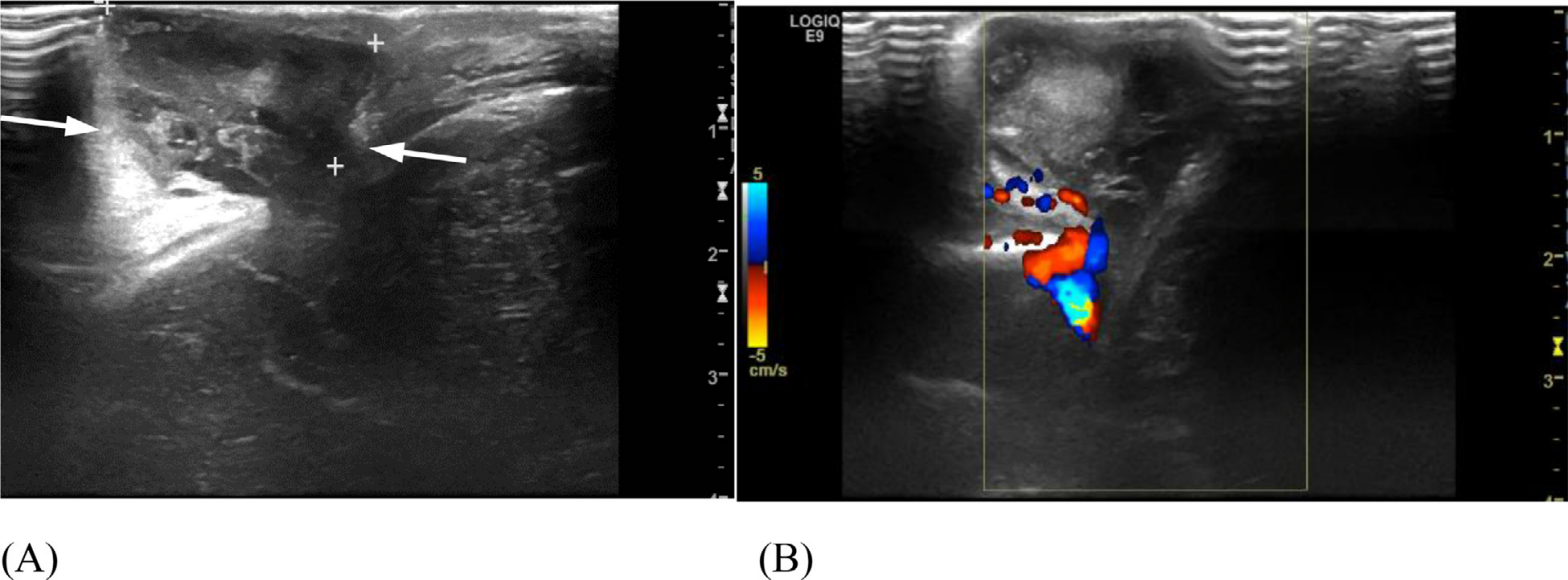
(Case 2) Head ultrasound without (A) and with (B) color Doppler demonstrates a skull defect and central echogenic debris herniating outside the calvarium. Color Doppler flow shows a falcine sinus extending into the extracalvarial mass, consistent with an atretic posterior cephalocele.

**Fig. 7:**
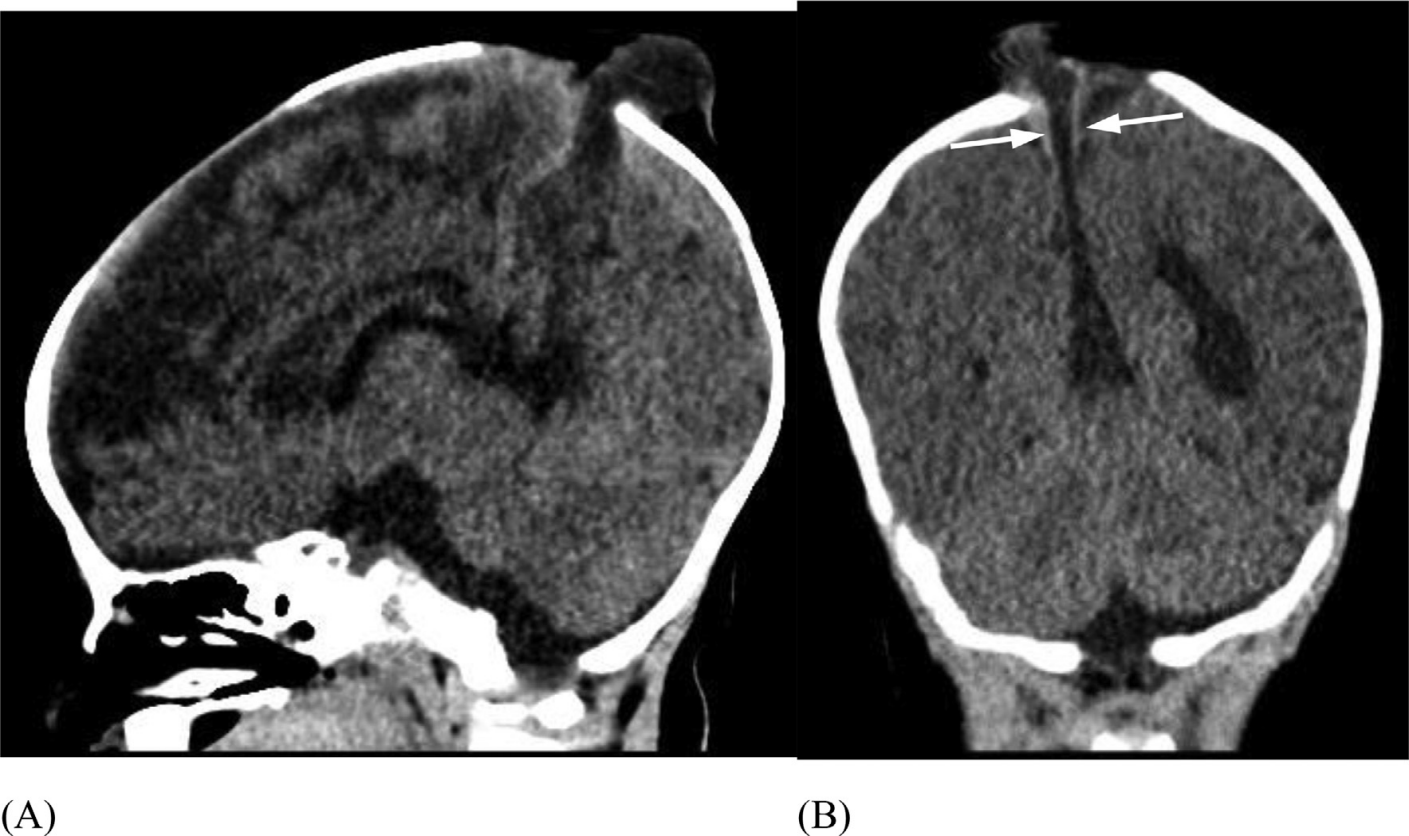
(Case 2) Sagittal midline CT (A) demonstrates the calvarial defect cranium bifidum with atretic parietal cephalocele. Coronal CT (B) demonstrates fenestration of the superior sagittal sinus at the atretic parietal cephalocele (arrows).

**Fig. 8:**
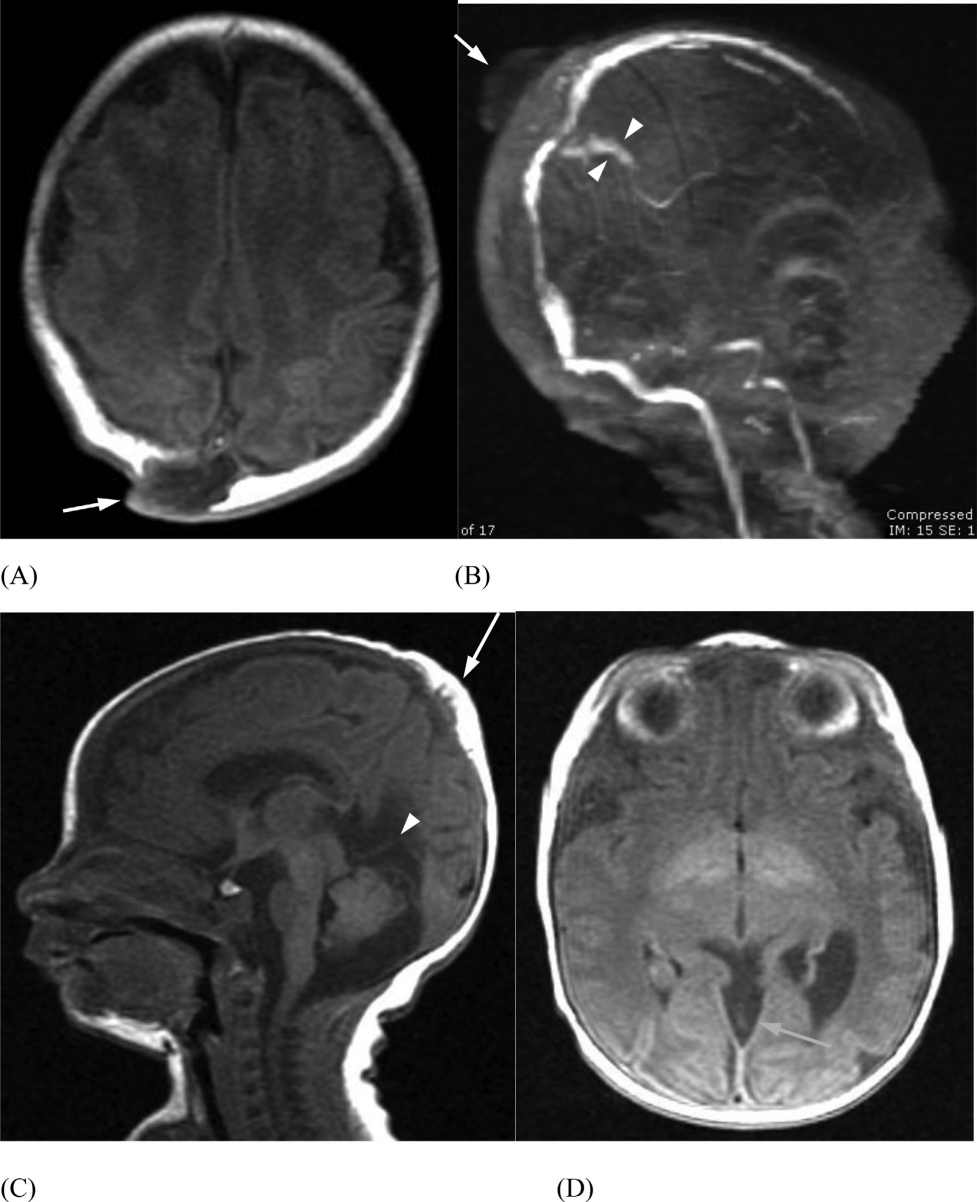
(Case 2) (A) Axial T1 demonstrates the atretic parietal cephalocele (APC) immediately posterior to the superior sagittal sinus (white arrow). (B) MRV demonstrates persistent vertical falcine sinus (arrowheads) extending superiorly to the APC (white arrow). (C) Sagittal T1 shows prominence of the superior cerebellar cistern, peaking of the tentorium (arrowhead) and the atretic parietal cephalocele (white arrow). Axial MRI (D) demonstrates an enlarged temporal horn of the left lateral ventricle and a prominent peri-pineal recess (blue arrow).

A baby boy was born at term by normal vaginal delivery with neonatal jaundice and a 2 cm midline, parietal, skin-covered, violaceus, non-pulsatile mass (Fig. 5). Head US demonstrated an APC (Fig. 6). Confirmatory follow-up head CT demonstrated a persistent falcine sinus extending into the 2 cm APC, which protruded through a 2 cm cranium bifidum defect (Fig. 7). Subsequent MRI revealed a stenotic but patent sagittal sinus with a prominent persistent falcine sinus, as well as tentorial “beaking” and a prominent peri-pineal recess (Fig. 8).

At 3 months old, the mother reported a possible “leak,” so the APC was excised earlier than initially planned. The APC was opened following resection and was found to be free of any structures. Its stalk was about 5 mm in external diameter and its lumen was very tiny. The patient tolerated the procedure well. At 3 years old, the patient underwent a cranioplasty to repair his skull defect. The patient also has a history of aortic root dilation, autism, and strabismus associated with chromosome 7q11.23 duplication syndrome.

### Case 3: Sinus thrombosis

**Fig. 9:**
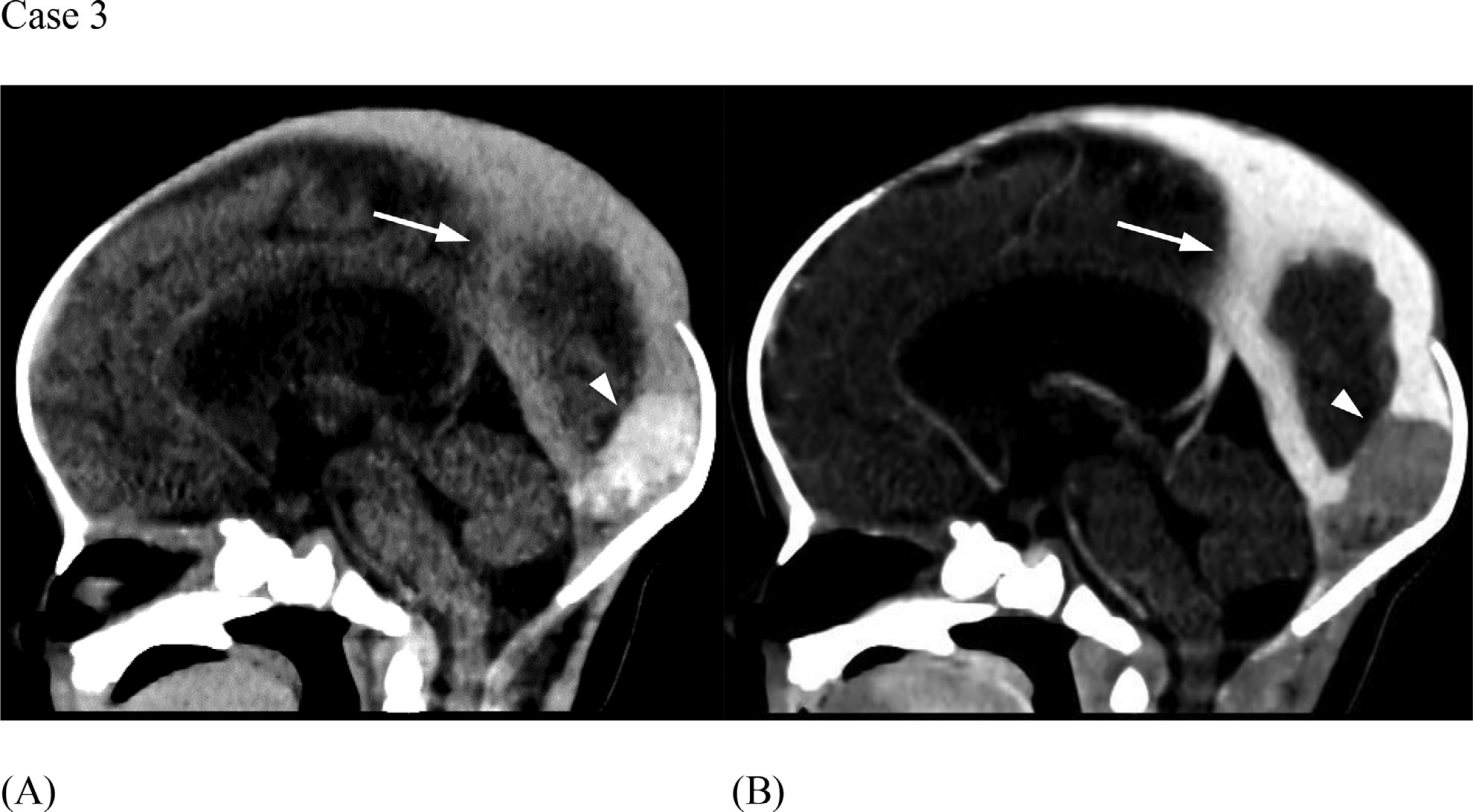
(Case 3) Sagittal CT without contrast (A) and with contrast (B) demonstrate persistent falcine sinus (arrows) and superior sagittal sinus leading to torcular thrombus (arrowheads), likely formed due to irregular flow dynamics.

**Fig. 10:**
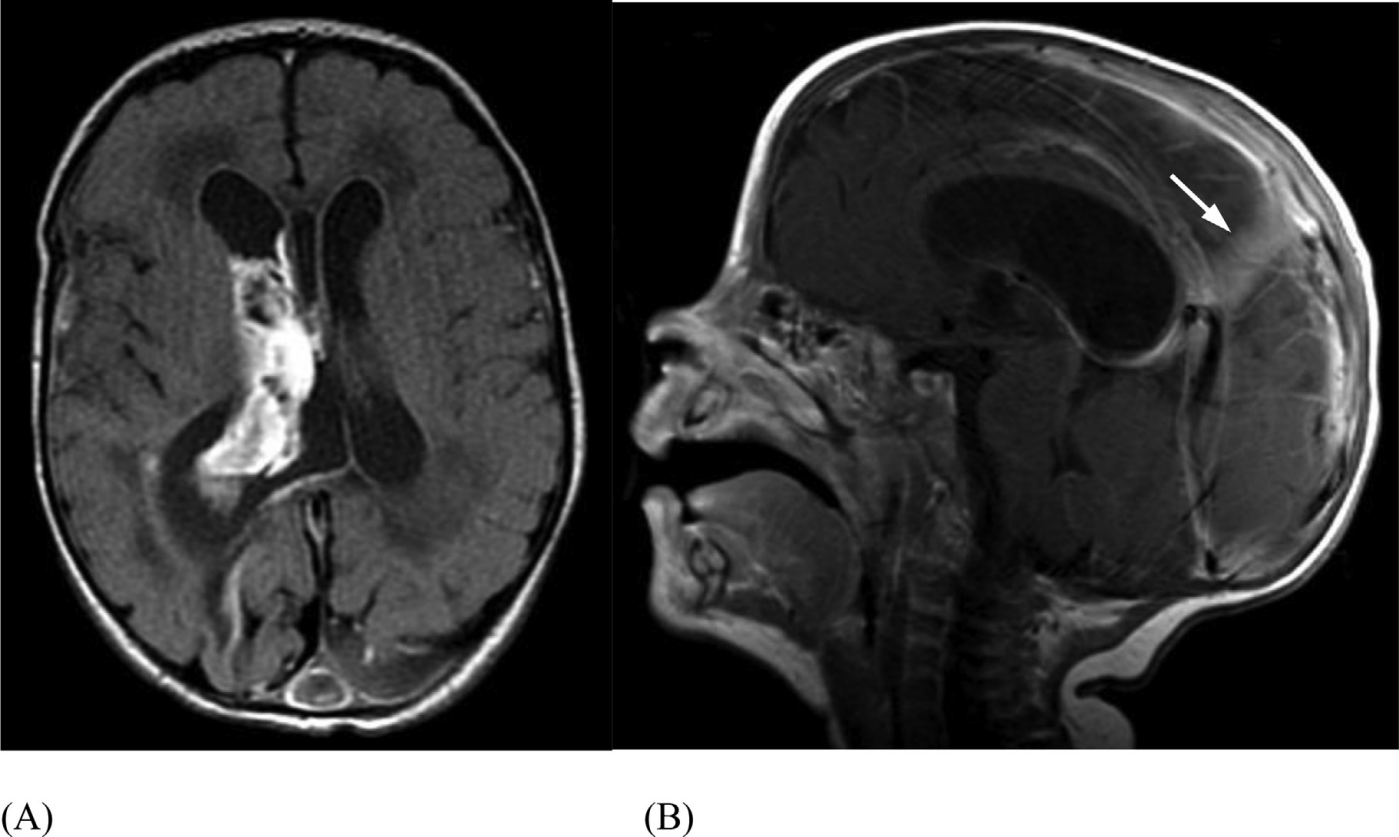
(Case 3) Axial T1 MR post-gadolinium (A) demonstrates the grade 3 germinal matrix hemorrhage. Sagittal T1 MR post-gadolinium (B) shows interval resolution of thrombus post-recanalization. There is persistent but decreased size and flow in falcine sinus (arrow).

A baby boy was born at term via C-section for breech presentation. He was tachypneic with a “high pitched cry” shortly after birth. Out of concern for sepsis, a diagnostic lumbar puncture was performed demonstrating gross blood. Neonatal head US evaluation revealed a large falcine sinus. At approximately 36 hours of life, a follow up head CT (Fig. 9) and brain MRI (Fig. 10) demonstrated a grade 3 germinal matrix hemorrhage and a thrombosis involving posterior inferior aspect of the superior sagittal sinus and torcula extending into the right transverse sinus. Anticoagulation with enoxaparin (Lovenox) was initiated and titrated. Lovenox was administered via an indwelling catheter for 2 months, at which point the patient's mother began administering the Lovenox herself without complications. At the time of publication, the patient was meeting all developmental milestones.

## Discussion

### Case 1: Vein of Galen arterial malformation (VGAM)

**Fig. 11:**
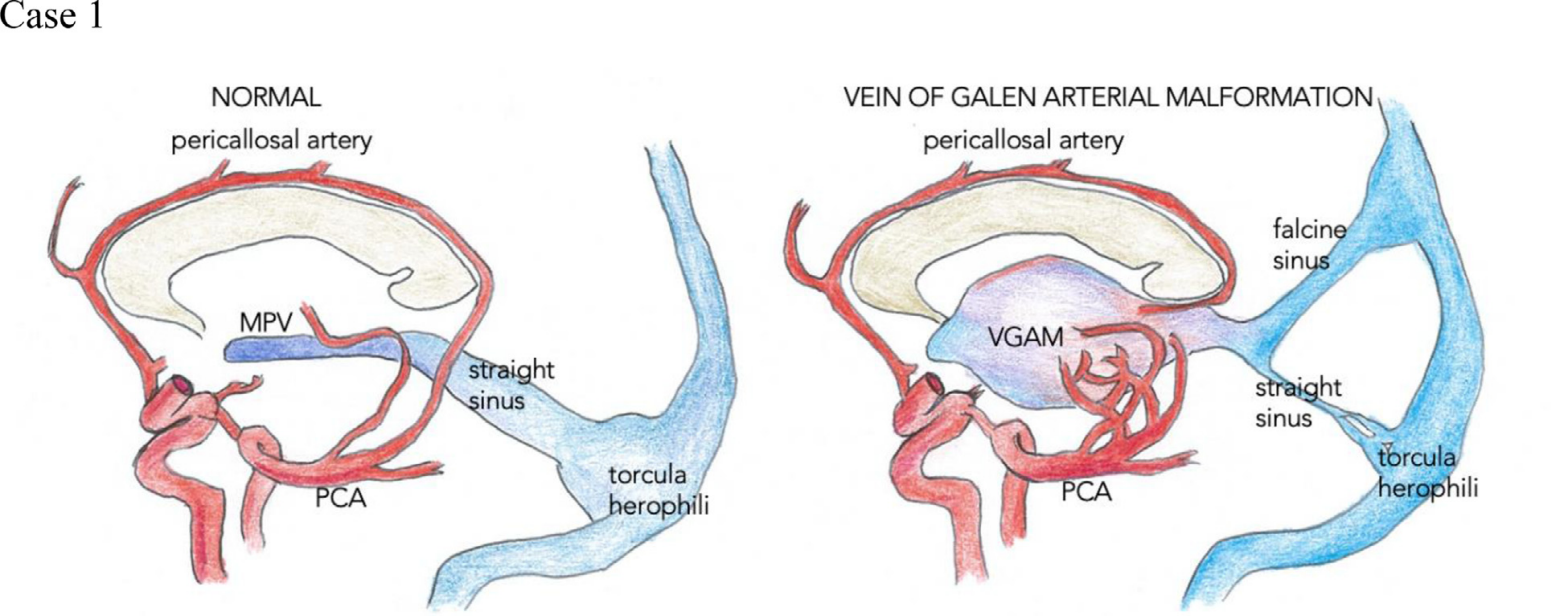
(Case 1) Vein of Galen arterial malformation (VGAM). MPV, median prosencephalic vein; PCA, posterior cerebral artery.

The posterior branches of the pericallosal arteries normally anastomose with the distal branches of the posterior cerebral arteries before birth. In VGAMs, these anastomoses degenerate, leading to an enlarged and malformed MPV (Fig. 11). The decreased resistance associated with the enlarged MPV often leads to severe high-output heart failure in the neonatal period. It has been reported in 1 in 25,000 live births [4]. The most common clinical presentations in children are failure to thrive, hydrocephalus, or heart failure [5]. In neonates, brain ischemia and multi-organ dysfunction secondary to hypoperfusion are most common [6]. Cerebral angiography is the diagnostic tool of choice, but computed tomography or magnetic resonance imaging can aid in diagnosis [7]. Endovascular therapy is the preferred treatment, which can reduce cardiovascular complications by decreasing blood flow through the malformation. The mortality rate is between 15% and 36% [8]. With appropriately timed treatment, most patients can survive and develop normally [4].

### Case 2: Atretic parietal cephalocele APC

Cephaloceles are rare herniations of intracranial structures through a congenital bone defect. They have an incidence of between 1 in 3500 and 1 in 5000 live births. Of these, 37.5% are atretic cephaloceles which are cephaloceles comprised of dura, fibrous tissue, and dysplastic brain tissue [9]. As of 2012, only 59 cases of AC have been reported in children and only 2 in adults in the literature [10]. Similar to the findings of Hsu et al, this APC contained a persistent falcine sinus remnant, suggestive that it may be on a spectrum of disease with sinus pericranii, (abnormal communication between intracranial dural sinuses and extracranial venous structures) as an alternative pathway of venous drainage in the prenatal brain [11]. APCs can be readily diagnosed on imaging, the best of which is magnetic resonance imaging. With surgical excision, the prognosis of APCs is generally good [12].

### Case 3: Sinus thrombosis

Neonatal cerebral venous sinus thrombosis is rare, and associated with comorbid conditions, 62% of the time, including dehydration (26%), sepsis (7%), cardiac defects (26%), and meningitis (10%) [13]. Maternal risk factors, such as hypercoagulability in preeclampsia, are also thought to contribute, however the literature is unclear. Unlike arterial strokes, CVTs occur more frequently in young adults and children. In this instance, there was no associated comorbidity, which is the case approximately 38% of the time [13]. Similar to a previous report on straight sinus thrombosis in an infant, this case of sinus venous thrombosis was thought to cause irregular flow dynamics leading to recanalization of the falcine sinus [14].

## Conclusion

The persistent falcine sinus may be associated with a diverse array of anomalies and is not an uncommon incidental finding. Although commonly seen with VGAM, arteriovenous malformations, absent corpus callosum, osteogenesis imperfecta, or Chiari II malformations, in this study, it was seen with VGAM, atretic parietal cephalocele, malformations of cortical development, and sinus thrombosis. We highlight the need to understand anatomic variations and anomalies in the cerebral venous system to ensure the proper diagnosis and treatment of the associated pathology.

## Patient consent

Appropriate informed written consent has been obtained for this publication.
